# The Warburg effect modulates DHODH role in ferroptosis: a review

**DOI:** 10.1186/s12964-022-01025-9

**Published:** 2023-05-05

**Authors:** Alvan Amos, Alex Amos, Lirong Wu, He Xia

**Affiliations:** 1 Department of Radiation Oncology, Jiangsu Cancer Hospital & Jiangsu Institute of Cancer Research & The Affiliated Cancer Hospital of Nanjing Medical University, 42 Baiziting Road, Nanjing, 210009 China; 2 Department of Biochemistry, Faculty of Science, Kaduna State University, PMB 2339 Tafawa Balewa Way, Kaduna, Nigeria; 3 Department of Pharmacology, Faculty of Pharmaceutical Sciences, Ahmadu Bello University Zaria, Zaria, Nigeria

**Keywords:** Mitochondria, Ferroptosis, Dihydroorotate dehydrogenase, Ribonucleotide reductase, Electron transport chain, Superoxide anion

## Abstract

Ferroptosis is an iron-dependent regulated cell death that suppresses tumor growth. It is activated by extensive peroxidation of membrane phospholipids caused by oxidative stress. GPX4, an antioxidant enzyme, reduces these peroxidized membrane phospholipids thereby inhibiting ferroptosis. This enzyme has two distinct subcellular localization; the cytosol and mitochondria. Dihydroorotate dehydrogenase (DHODH) complements mitochondrial GPX4 in reducing peroxidized membrane phospholipids. It is the rate-limiting enzyme in de novo pyrimidine nucleotide biosynthesis. Its role in ferroptosis inhibition suggests that DHODH inhibitors could have two complementary mechanisms of action against tumors; inhibiting de novo pyrimidine nucleotide biosynthesis and enhancing ferroptosis. However, the link between mitochondrial function and ferroptosis, and the involvement of DHODH in the ETC suggests that its role in ferroptosis could be modulated by the Warburg effect. Therefore, we reviewed relevant literature to get an insight into the possible effect of this metabolic reprogramming on the role of DHODH in ferroptosis. Furthermore, an emerging link between DHODH and cellular GSH pool has also been highlighted. These insights could contribute to the rational design of ferroptosis-based anticancer drugs.

**Video Abstract**

**Supplementary Information:**

The online version contains supplementary material available at 10.1186/s12964-022-01025-9.

## Background

Oncogenic mutations significantly contribute to tumorigenesis. An example is the mutation that affects Ras superfamily of small GTPases [1]. It was discovered that two chemical compounds; erastin and Ras-selective lethal compound 3 (RSL3) selectively induce cell death in Ras-mutant cancers. This cell death mechanism lacks the classical molecular and biochemical features of apoptosis. Rather, it was characterized by a high cellular level of oxidative stress markers and also susceptible to inhibition by iron chelation or blocking cellular iron uptake [2]. To capture its iron dependency, this novel cell death mechanism was termed “ferroptosis” [2]. RSL3 inhibits glutathione peroxidase 4 (GPX4), an antioxidant enzyme having two distinct subcellular localization; the cytosol and the mitochondria [3]. This enzyme protects membrane phospholipids from peroxidation [4]. Erastin is an inhibitor of cystine-glutamate antiporter (xCT), a transmembrane protein that imports cystine into the cell [5]. Cystine is then reduced to cysteine, the rate-limiting amino acid in the biosynthesis of reduced glutathione (GSH) [5]. GSH serves as a cofactor for GPX4.

Following its first description, ferroptosis has been implicated in the pathogenesis of cancer, neurological disorders, coronary heart diseases, liver, and kidney diseases [6]. However, most of the studies focused on the involvement of ferroptosis in cancer, and a recent finding is the contribution of dihydroorotate dehydrogenase (DHODH) to ferroptosis inhibition. It was reported that DHODH works along with mitochondrial GPX4 to inhibit ferroptosis [3]. DHODH is the rate-limiting enzyme in de novo pyrimidine nucleotide biosynthesis located in the mitochondria. It links de novo pyrimidine nucleotide biosynthesis to the electron transport chain (ETC) at complex III through Coenzyme Q (ubiquinone) pool [7]. This suggests that in addition to suppressing tumor growth by inhibiting pyrimidine biosynthesis, DHODH inhibitors could have a second mechanism of action that is, enhancement of ferroptosis.

In contrast to normal cells, which rely on mitochondrial oxidative phosphorylation to generate adenosine triphosphate (ATP), most cancer cells instead use aerobic glycolysis also known as the Warburg effect [8]. This metabolic reprogramming could be a biochemical adaptation that supports the biosynthetic requirements of rapidly proliferating cells [9]. Since nucleic acid synthesis is a key biosynthetic requirement of cancer cells, the metabolic reprogramming associated with the Warburg effect could enhance its efficiency. It is important to note that the Warburg effect affects the functioning of mitochondrial ETC which is linked to the de novo pyrimidine nucleotide biosynthesis by DHODH at complex III [7]. Given the role of the ETC in the generation of oxidative stress-inducing reactive oxygen species (ROS) and the involvement of DHODH in ETC function; we reviewed previous studies to understand the possible effect of the Warburg effect on the role of DHODH in ferroptosis.

### The electron transport chain and ferroptosis

The ETC is a series of inner mitochondrial membrane protein complexes and biochemical mobile electron carriers which facilitate the movement of electrons extracted from fuel molecules to oxygen to form water [10]. Electron movement along the ETC occurs with a concurrent translocation of protons into the intermembrane space to generate a proton gradient. The proton gradient is dissipated when H^+^ re-enters the mitochondrial matrix through ATP synthase also known as complex V [11]. The other protein complexes are complex I, complex II, complex III, and complex IV. Electron leakage along the ETC produces O_2_^−^. This primarily occurs at complex I or complex III [12].

**Fig. 1:**
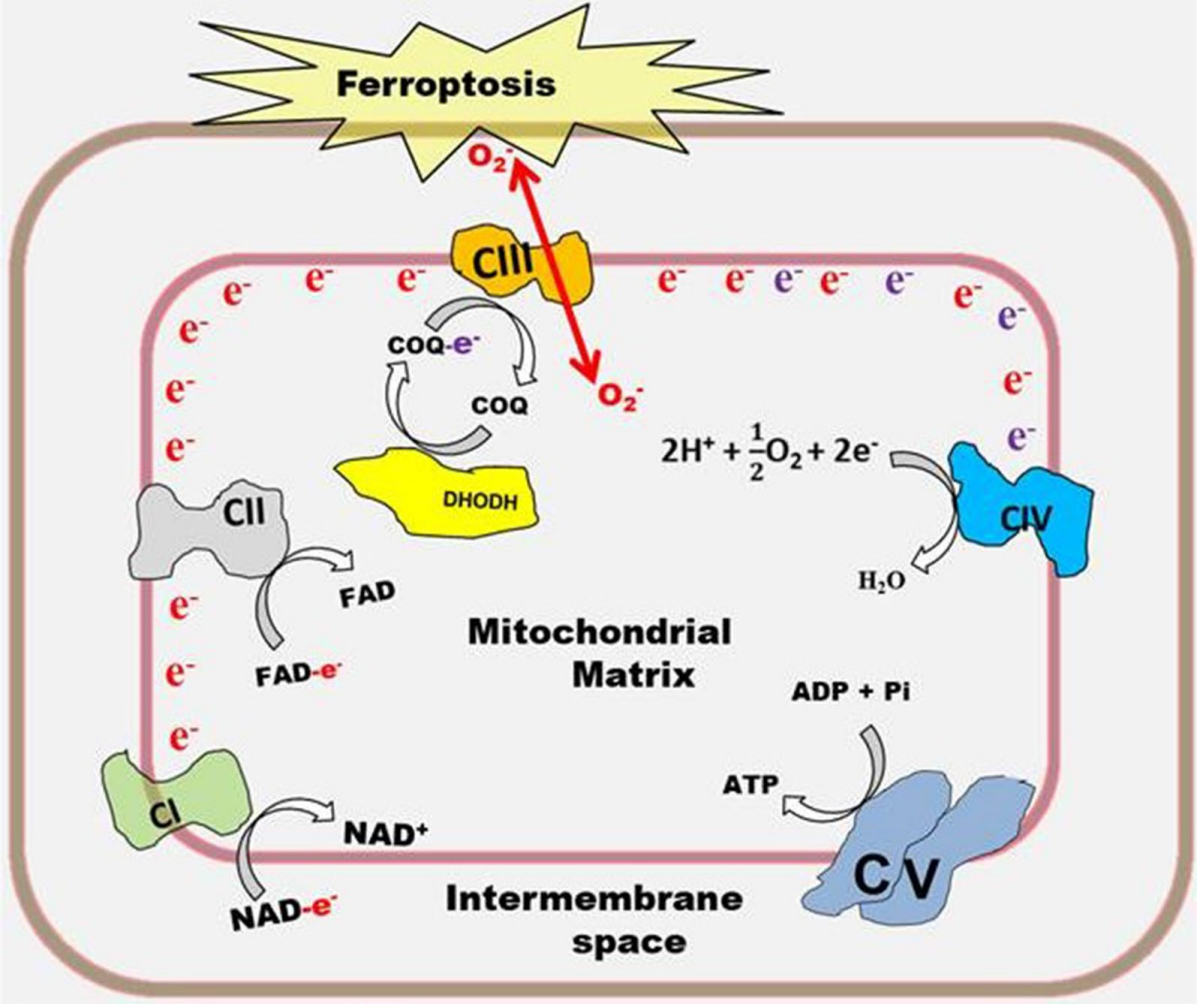
Contribution of ETC to ferroptosis induction. Electrons shuttled into the ETC come from NADH (represented as NAD-e^−^), FADH_2_ (represented as FAD-e^−^), and CoQ10H_2_ (represented as COQ-e^−^). Complex III (CIII)-generated O_2_^−^ has been shown to play a key role in the induction of ferroptosis activated by cysteine deprivation

It was reported that O_2_^−^ produced by complex III of the ETC plays a pivotal role in the induction of ferroptosis by cysteine deprivation [13]. The study revealed that S3QEL, a molecule that suppresses O_2_^−^ production by complex III and not S1QEL which suppresses O_2_^−^ generation by complex I could protect mouse hepatoma Hepa 1–6 cells from lipid peroxidation and ferroptosis. In addition, S3QEL was able to inhibit ferroptosis in xCT-knockout mouse-derived embryonic fibroblasts [13]. The xCT knock out (KO) causes cysteine deficiency which inhibits glutathione biosynthesis. This study suggests that the functionality of complex III of the ETC may be required for pharmacological induction of ferroptosis. The ability of complex III-generated O_2_^−^ to induce lipid peroxidation could be due to its bi-directional release both into the mitochondrial matrix and the intermembrane space (Fig. 1) as opposed to complex I-generated O_2_^−^ which is only released into the mitochondrial matrix [12].

It was earlier reported that the biochemical mechanism for the induction of ferroptosis by erastin is mitochondrial membrane potential hyperpolarization [14]. However, this study did not report the dynamics of O_2_^−^ generation by the ETC under experimental conditions. It is conceivable that the membrane potential hyperpolarization observed during the study was linked to ferroptosis induction due to a positive correlation with O_2_^−^ generation by the ETC. This view is supported by the failure of complex I and II inhibitors to sustainably suppress ferroptosis during the study, suggesting that provided electrons pass through complex III and IV, ferroptosis could be activated. The generation of O_2_^−^ by complex III could be a more plausible hypothesis explaining the biochemical mechanism underlying ferroptosis induction by erastin. This is because of the well-established relationship between ferroptosis and oxidative stress.

A third study that reported the biochemical mechanism underlying the induction of ferroptosis by erastin also implicated O_2_^−^ generation [15]. However, this study concluded that the ETC is not involved in erastin-induced ferroptosis but rather, the pyruvate dehydrogenase complex-catalyzed reaction. This is because treating HT1080 cells with Antimycin A or FCCP was found to have no significant effect on the cell’s sensitivity to erastin as opposed to the genetic inhibition of E1 or E3 subunits of pyruvate dehydrogenase complex. This study used changes in the E_50_ value of erastin as indicative of ferroptosis sensitivity. A possible drawback to the validity of the conclusion dissociating ETC from erastin-induced ferroptosis is the absence of data showing that the cells actually died as a result of ferroptosis. This data may be important because Antimycin and FCCP can interact with erastin to modify its pharmacological effect. In addition, although the decrease in oxygen consumption rate (OCR) observed as a result of Antimycin treatment is expected to correlate with decreased electron flow through the ETC resulting in decreased O_2_^−^ production, a previous study has reported an increase in cellular O_2_^−^ level in response to Antimycin treatment [16]. If this happens to be the case in this study, although a significant difference in OCR was observed between the Antimycin and FCCP treated group, there may be no significant difference in cellular O_2_^−^ levels. Therefore, the use of a different pharmacological agent like S3QEL or a genetic approach to the inhibition of complex III of the ETC may tell a different story.

### DHODH and ferroptosis

**Fig. 2:**
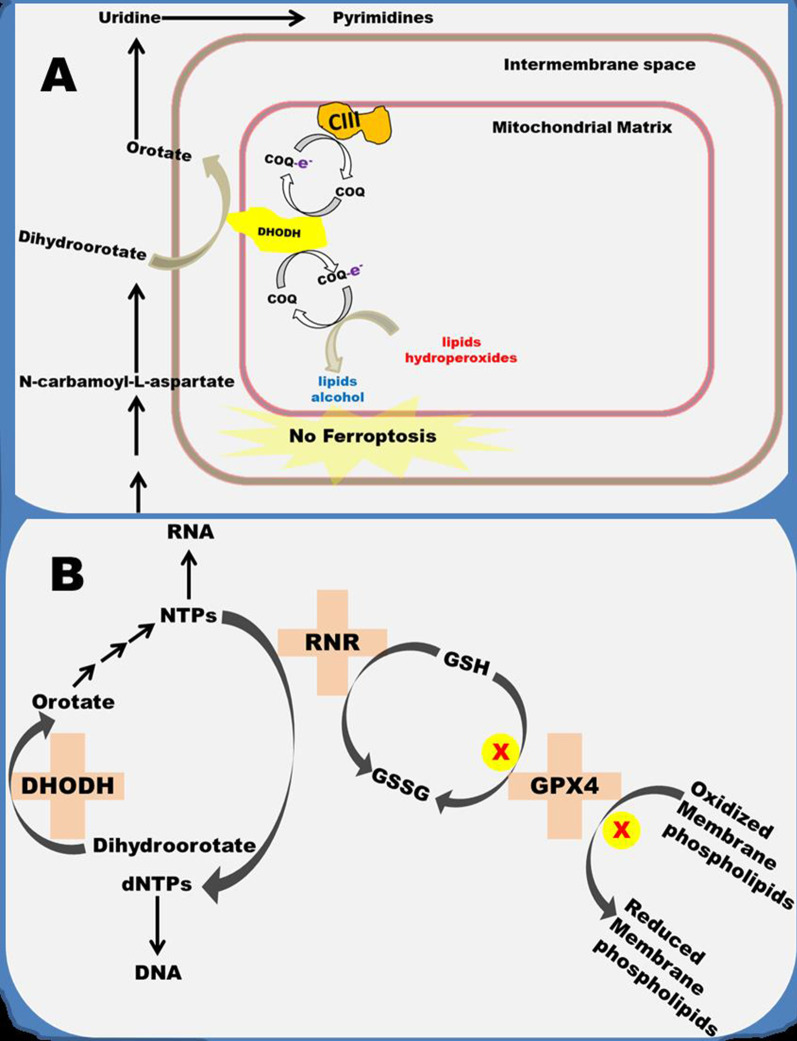
**A** Ferroptosis inhibition by DHODH. DHODH-generated Ubiquinol (COQ-e-) reduces mitochondrial membrane phospholipids hydroperoxides to lipids alcohol, thereby inhibiting the onset of ferroptosis. **B** Ferroptosis induction by DHODH. DHODH-catalyzed reaction indirectly reduces the cellular level of GSH. This is because the nucleoside triphosphates (NTPs) it produces are required for RNR-catalyzed reaction which uses GSH as a cofactor. This can inhibit GPX4 via GSH depletion

DHODH functions along with mitochondrial GPX4 to reduce peroxidized membrane phospholipids [17]. Metabolomics screening following the treatment of cancer cells with GPX4 inhibitors resulted in a significant depletion of *N*-carbamoyl-L-aspartate and the accumulation of uridine. *N*-carbamoyl-L-aspartate is a de novo pyrimidine nucleotide biosynthesis intermediate located upstream of DHODH while uridine is an intermediate downstream of DHODH. These changes suggest that GPX4 inhibition enhanced the conversion of *N*-carbamoyl-L-aspartate to uridine. Given that DHODH is the rate-limiting enzyme of this pathway, it is conceivable that the flow of metabolites can only increase if DHODH activity is upregulated. Supplementing the culture medium with dihydroorotate, a substrate for DHODH, suppressed ferroptosis activated by GPX4 inhibition while supplementing the medium with uridine, the end product of the pathway, enhanced ferroptosis induced by GPX4 inhibition. These observations suggested a complementary role between GPX4 and DHODH in ferroptosis inhibition, a hypothesis that was later confirmed during the study [17]. Further investigations revealed that the synergy is between mitochondrial GPX4 and DHODH. A mechanistic study showed that the lipophilic antioxidant, ubiquinol (CoQ10H_2_), produced during DHODH-catalyzed conversion of dihydroorotate to orotate is the anti-ferroptosis molecule. This mitochondria-localized ferroptosis defense mechanism has its cytosolic counterpart involving the conversion of ubiquinone (CoQ10) to CoQ10H_2_ by ferroptosis suppressor protein 1 (FSP-1) [18]. Whether other ubiquinol-generating reactions within the mitochondria inhibit ferroptosis is yet to be investigated. This biochemical mechanism linking DHODH with ferroptosis is represented in Fig. 2A.

A second link between DHODH and ferroptosis is found within the deoxyribonucleic acid (DNA) biosynthetic pathway. Nucleosides triphosphates (NTPs), which are the precursors for deoxynucleosides triphosphates (dNTPs) used for DNA synthesis, are derived from purines and pyrimidines [19]. Given that during DNA synthesis, purines pair with pyrimidines, insufficient pyrimidines could terminate the base-pairing process. The de novo pyrimidine biosynthetic pathway plays an important role in rapidly proliferating cells. This is due to the need to meet the cell’s high nucleic acid demand. Since DHODH catalyzes the rate-limiting step in de novo pyrimidine biosynthesis, its inhibition could decrease the cellular levels of pyrimidines. Low cellular levels of pyrimidines will slow down ribonucleotide reductase (RNR)-catalyzed reaction which converts NTPs to dNTPs. This assertion is supported by a significant decrease in dNTPs observed following DHODH inhibition [20]. A recent study reported that ferroptosis could be suppressed by inhibiting RNR [21]. This is because RNR uses GSH as a cofactor. When RNR is inhibited, the cellular GSH pool increases. This becomes available to GPX4 to use for reducing peroxidized membrane phospholipids. Therefore, DHODH depletion could enhance the efficiency of GPX4 via inhibiting RNR. However, DHODH depletion may not inhibit ferroptosis in cells with low GPX4 expression via this biochemical mechanism. This biochemical mechanism linking DHODH with ferroptosis is represented in Fig. 2B.

### The role of DHODH and complex III in tumor growth

Tumor cells are characterized by rapid growth and division. As a result, most of the biosynthetic precursors are synthesized de novo [22]. To sustain growth in tumors, cell division must progress rapidly. This cannot occur when biosynthetic precursors required for the S phase of the cell cycle are insufficient. The S phase facilitates chromosome doubling that precedes cell division. This phase in tumors will require a large supply of pyrimidines that can only be made available when synthesized de novo. DHODH, the rate-limiting enzyme for de novo pyrimidine nucleotide biosynthesis, links this pathway to complex III of the ETC [23]. The reaction mechanism for DHODH involves the reduction of ubiquinone (CoQ10) to CoQ10H_2_. CoQ10 is a mobile electron carrier that accepts electrons at complexes I and II of the ETC and transfers them to complex III. However, DHODH-generated CoQ10H_2_ donates its electrons directly to complex III [23]. Therefore, inhibition of complex III will not only halt electron flow through the ETC but could also inhibit DHODH-catalyzed conversion of dihydroorotate to orotate, thereby shutting down de novo pyrimidine nucleotide biosynthesis. This could result in cell cycle arrest and the inhibition of tumor growth. Inhibition of complex III of mitochondrial ETC by antimycin A was able to inhibit proliferation and promote cellular differentiation in acute myeloid leukemia (AML) cells through a DHODH inhibition-mediated mechanism [24].

Another study reported impaired tumor growth due to a dysfunctional complex III of the ETC. The biochemical mechanism underlying this growth inhibition involves the failure of complex III to re-oxidize CoQ10H_2_ back to CoQ10 rather than the failure of electrons to flow through the ETC [25]. During the study, ectopic expression of *Ciona intestinalis* alternative oxidase (AOX) which oxidizes CoQ10H_2_ to CoQ10 restored the lost tumor growth. In addition, a functional complex III and IV of the ETC alone sufficiently restored tumor growth of respiration-deficient cancer cells through the re-activation of de novo pyrimidine nucleotide biosynthesis [26]. These studies provide mechanistic insight into a previous observation where the acquisition of mitochondrial DNA (mtDNA) via horizontal transfer of whole mitochondria from host cells to mtDNA-deficient cancer cells restored lost tumorigenicity [27, 28]. The transferred host mitochondria could have facilitated the rate-limiting step in de novo pyrimidine nucleotide biosynthesis. Also, these findings are an addition to the accumulating evidence against complete mitochondrial damage as the biochemical basis for aerobic glycolysis in tumors. During aerobic glycolysis also known as the Warburg effect, some aspects of mitochondrial function could be inefficient; however, the ETC-linked DHODH-catalyzed rate-limiting step for de novo pyrimidine nucleotide biosynthesis should not be affected. This conclusion is further supported by the need for a functional complex III for hepatitis E virus replication [29]. In fact, disruption in certain aspects of mitochondrial function during aerobic glycolysis, in addition to conferring other tumorigenic advantages, appears to be aimed at optimizing de novo pyrimidine nucleotide biosynthesis. This optimization could be due to the high nucleic acid demand during the S-phase of the cell cycle [30]. For example, hypoxia can slow down the movement of electrons through the ETC due to insufficient oxygen molecules that can serve as terminal electron acceptors. A defect in complex I and II of the ETC associated with the Warburg effect [31, 32] could enable more efficient coupling of mitochondrial oxygen consumption to the conversion of dihydroorotate to orotate in an oxygen-deficient intracellular environment predominant in solid tumors.

The importance of de novo pyrimidine nucleotide biosynthesis for the proliferation of human Jurkat leukemic T cells is indirectly substantiated by a metabolic reprogramming activated by complex I inhibition. When the ETC is fully functional, glutamate–oxaloacetate transaminase 1 (GOT1) converts aspartate to oxaloacetate and α-ketoglutarate to glutamate concurrently. This reaction is part of the cytosolic portion of the malate-aspartate shuttle that transfers NADH produced during glycolysis from the cytosol into the mitochondria. The mitochondrial matrix portion of this shuttle comprises malate dehydrogenase 2 (MDH2) and glutamate–oxaloacetate transaminase 2 (GOT2). These enzymes synthesize aspartate which is transported to the cytosol for use by GOT1 and other enzymes. However, complex I inhibition inhibits the regeneration of NAD^+^ by the ETC. This leads to the inhibition of the mitochondrial matrix portion of the shuttle. Under this condition, GOT1-catalyzed reaction reverses, thereby producing aspartate from the oxaloacetate produced by ATP citrate lyase-catalyzed reaction in the cytosol. This citrate comes from the reductive decarboxylation of glutamine in the mitochondrial matrix [33]. Following complex I inhibition, these cells were found to rely on GOT1-dependent aspartate biosynthesis for proliferation [33].

A defect in complex I of the ETC rewires the malate-aspartate shuttle, shutting down GOT2-dependent aspartate biosynthesis while activating GOT1-dependent aspartate biosynthesis which is linked to the reductive decarboxylation of glutamine. This could be the reason why tumors which usually have a defective ETC due to the Warburg effect are heavily reliant on glutamine metabolism for proliferation [34, 35]. It is important to note that during the study, experimental data suggested that a significant fraction of the aspartate synthesized by GOT1 goes to purine and pyrimidine nucleotide biosynthesis [22]. Given that, GOT1-dependent aspartate biosynthesis could only support cell proliferation if DHODH and complex III are functional. The coupling of DHODH-catalyzed reaction to complex III of the ETC [36] suggests that reductive decarboxylation of glutamine which supplies the oxaloacetate used by GOT1 could result in the generation of ATP in the ETC through DHODH-mediated shuttling of CoQ10H_2_ to complex III [37].

High extracellular lactate characteristic of the Warburg effect induces reductive decarboxylation of glutamine in breast cancer cells [38] and tumor hypoxia which can also induce the Warburg effect making aspartate become a limiting metabolite for cell proliferation [39]. Aspartate was found to be limiting for cell proliferation because of its role in de novo purine and pyrimidine nucleotide biosynthesis. In pyrimidine biosynthesis, aspartate contributes three carbon atoms during the production of uridine monophosphate (UMP) and thymidine monophosphate (TMP) [39]. Under hypoxia and ETC dysfunction, 50 to 80% of orotate, dTMP, and UMP in solute carrier family 1 member 3 (SLC1A3)-expressing cells were derived from extracellularly imported aspartate. Thus suggests that hypoxia could reduce the intracellular concentration of pyrimidines synthesized de novo by more than 50% unless an adaptive metabolic rewiring occurs to supply aspartate. This study suggests a relationship between defective complex I, GOT1-facilitated aspartate biosynthesis, and nucleotide biosynthesis.

Coupling of de novo pyrimidine nucleotide biosynthesis to the ETC could be a strategy for meeting the high energy demand for pyrimidine biosynthesis when metabolic rewiring occurs that shuts down part of the energy-generating mitochondrial catabolism in favor of energy-demanding nucleotide biosynthesis. This is because DHODH-facilitated electron transfer to complex III and IV could result in pumping protons into the intermembrane space thereby generating some transmembrane electrochemical potential that could be used for ATP synthesis by complex V [40]. This notion is supported by the efficient induction of mitochondrial membrane potential depolarization by DHODH inhibitors from two distinct chemical series [41]. Findings from another study suggest that preceding UVB-Induced primary skin tumor formation, a metabolic rewiring occurs that shuts down glycolysis and the TCA cycle (thereby shutting down complex I and II) while upregulating the distal part of the ETC which includes a heightened DHODH activity [42]. In this study, DHODH was found to sustain ATP generation by the ETC.

**Fig. 3:**
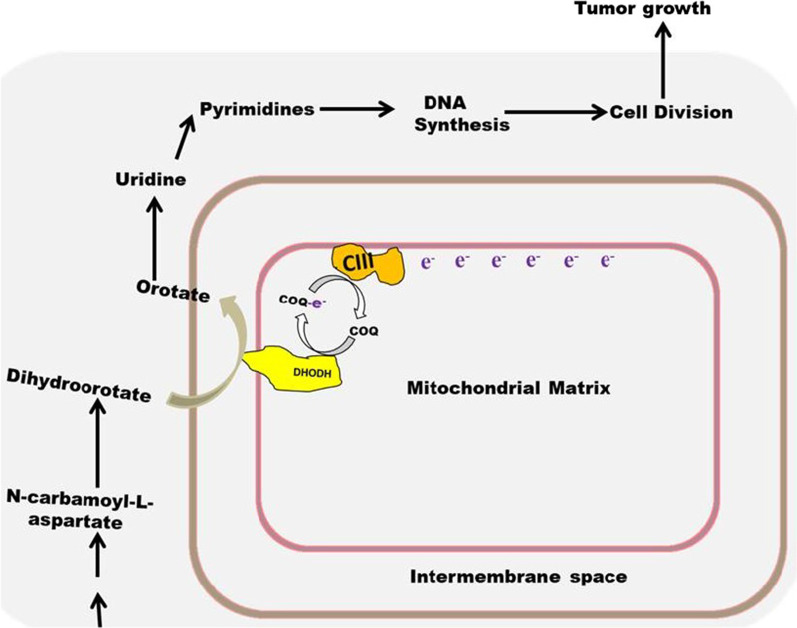
Role of DHODH and complex III in tumor growth. DHODH-catalyzed reaction is the rate-limiting step in de novo pyrimidine nucleotide biosynthesis. It catalyzes the conversion of dihydroorotate to orotate used for nucleic acid biosynthesis. This reaction needs the activity of complex III of the ETC and it involves the conversion of ubiquinol (COQ-e-) to ubiquinone (COQ)

Other defects in mitochondrial function that could selectively shut down complex I and II while preserving DHODH-catalyzed reaction and complex III include a tumor-associated defect in three enzymes of the TCA cycle [43]. These enzymes are succinate dehydrogenase (SDH), fumarate hydratase (FH), and isocitrate dehydrogenase (IDH). Defects in these enzymes affect cellular ROS status in a way that induces oxidative stress and stabilizes HIF1-α. While a deficiency in SDH increases ROS levels that result in HIF-1*α* stabilization, IDH1 and IDH2 mutation lowers the level of GSH and NADPH thereby reducing the efficiency of GSH and NADPH-dependent antioxidant enzymes. However, to manifest the metabolic phenotype of SDH mutant tumors, additional inhibition of complex I is required [44]. The absence of complex I inhibition has been postulated to be the differentiating factor between neurodegeneration-linked SDH deficiency and tumor-linked SDH deficiency [44]. A functional complex I could support glutaminolysis and the malate-aspartate shuttle, two pathways that could transfer electrons to complex I thereby reducing the efficient coupling of DHODH-catalyzed reaction to the utilization of the limited oxygen available in a tumor hypoxic environment. The mutation in FH inhibits complex I through the succination of its Fe-S proteins and complex II through a feedback inhibition activated by fumarate [45]. Role of DHODH and complex III in tumor growth is schematically represented in Fig. 3.

### Warburg effect and its modulation of the role of DHODH in ferroptosis

Although DHODH complements mitochondrial GPX4 to inhibit ferroptosis, the prevalence of Warburg effect in tumors, indispensability of DHODH-catalyzed reaction for tumor growth and the role of ETC in O_2_^−^ generation and ferroptosis induction suggest that inhibiting DHODH in some tumors could result in ferroptosis inhibition. This could result in drug resistance. Solid tumors are characterized by oxidative stress [46]. As ROS inhibit complex I and II of the ETC and the TCA cycle, it stabilizes HIF1-α which upregulates glycolysis as an adaptive response for ATP generation [47]. HIF1-α is an important component of HIF-1, a transcription factor that targets key glycolytic enzymes [47]. Active HIF-1 complex is a heterodimer made up of an oxygen-sensitive HIF-1α subunit and a constitutively expressed HIF-1β subunit. The HIF-1α subunit has an oxygen-dependent degradation domain (ODD) which contains two regulatory prolyl residues; P402 and P564. Under normal cellular oxygen concentration, these amino acid residues become hydroxylated by HIF prolyl-hydroxylases (PHDs). HIF-1 prolyl-hydroxylation facilitates the proteasomal degradation of this transcription factor [48]. However, even in the presence of oxygen, studies have shown that HIF-1α can be stabilized by ROS produced by the ETC.

In a study to establish the molecular mechanism underlying the activation of HIF-1 by angiotensin-II in vascular smooth muscle cells, it was discovered that ROS produced by the ETC play a key role [49]. The use of mitochondrial ETC complex III inhibitors, stigmatellin, and myxothiazol were found to inhibit the accumulation of HIF-1 induced by angiotensin-II. A second approach used in the study involved suppressing ROS production through siRNA-mediated inhibition of Rieske Fe-S protein, an essential component of complex III. This equally resulted in the inhibition of HIF-1α accumulation. The use of SkQ1, an antioxidant targeting mitochondrial ETC also produced the same result. This suggests that O_2_^−^ generated by the ETC plays an important role in the stabilization of HIF-1α. Genetic and pharmacological inhibition of NADPH oxidase (NOX), a mitochondrial enzyme that produces O_2_^−^ failed to sustain HIF-1α accumulation under non-hypoxic conditions [49]. This study concluded that ROS generated by the mitochondria is responsible for blocking HIF-1α hydroxylation and its subsequent proteasomal degradation during Angiotensin II treatment in vascular smooth muscle cells. Stabilization of HIF-1 by oxidative stress induces the Warburg effect.

Another study using hepatoma cells also supports the involvement of oxidative stress in inducing the Warburg effect even in the presence of oxygen [50]. During this study, it was discovered that the ability of hypoxia to upregulate glycolysis is dependent on ROS. This is because, in the presence of ROS scavengers, hypoxia failed to upregulate glycolysis. Hypoxia could increase both ROS level and HIF1-α activity. However, when ROS level was reduced using the antioxidant α-Lipoic Acid, the expression of HIF1-α was downregulated. The study further revealed that increasing endogenous ROS generation by xanthine oxidase gene transfection or decreasing it using antioxidants or MnSOD transfection could modulate cellular glycolytic activity independent of hypoxia status. Another group used mitoubiquinone, a mitochondria-targeted antioxidant to confirm the involvement of mitochondrial ROS in the stabilization of HIF-1α [51]. Furthermore, data from a study designed to assess the limiting metabolites required for cell proliferation under hypoxia suggests that tumor hypoxia is sufficient to inhibit the ETC [39]. It should be noted that if this inhibition affects complex III, tumor growth could be suppressed.

PTEN-induced kinase 1 (PINK1) inhibits the Warburg effect by reducing mitochondrial ROS generation. It also suppresses the growth of glioblastoma [52] suggesting that the Warburg effect, which is characterized by defective ETC, supports tumor growth. PINK1 suppresses ROS and tumor growth through FOXO3a, a transcription factor that regulates SOD2. ROS-mediated stabilization of HIF1-α was observed in PINK1 knockout (KO) cells. This resulted in the upregulation of genes that shifted glucose metabolism to aerobic glycolysis [52]. However, although the M2 isoform of pyruvate kinase (M2-PK) required for glycolysis was also upregulated, it remained in the inactive dimeric form due to ROS-mediated oxidation of its cysteine residues. The inactive dimeric form of M2-PK caused the accumulation of phospho-metabolites above phosphoenolpyruvate (PEP) in the glycolytic pathway. These metabolites were channeled to biosynthetic reactions including nucleic acid biosynthesis [53].

NADH:ubiquinone oxidoreductase subunit A10 (NDUFA10) is a key component of complex I of the ETC. PINK1 loss of function mutation affects complex 1 activity through the failure of serine 250 of NDUFA10 to become phosphorylated [54]. This is because a phosphorylated NDUFA10 is required for the reduction of ubiquinone by complex I [54]. This suggests that in addition to shifting glucose metabolism towards aerobic glycolysis through an oxidative stress-mediated stabilization of HIF-1, loss of PINK1 activity could affect the ETC by direct inhibition of complex I. The question now arises: between oxidative stress and the inhibition of complex I activity, which of the two plays the most important role in the induction of aerobic glycolysis? A study that assessed the effect of complex I deficiency and a defect in complex I assembly on the Warburg effect suggest that in the absence of ROS, a deficiency in complex I activity may not be sufficient to induce aerobic glycolysis [31]. Complex I is made up of the matrix arm and the membrane arm. The matrix arm is composed of two modules, the N (NADH binding) and Q (Quinone binding) module while the membrane arm comprises the P (Proton pumping) module. While the P module is encoded by mitochondrial DNA, the N and Q modules are encoded by nuclear DNA. These modules are built independently and later assembled together [55]. This study revealed that a defect in complex I assembly results in excessive ROS generation which resulted in the inhibition of pyruvate dehydrogenase in an AMPK-dependent manner.

Another mechanism through which mitochondrial ROS activates the Warburg effect involves the upregulation of mitochondrial uncoupling proteins (UCPs). Induction of oxidative stress in mouse skin tissues and embryonic fibroblast primary cell culture through SOD2 KO induced the Warburg effect. It was discovered that the oxidative stress-induced the uncoupling of the ETC to ATP synthesis which upregulated glycolysis to compensate for the reduction in ATP generated by the mitochondria [56]. A mechanistic study revealed that SOD2 deficiency caused the nuclear receptor; peroxisome proliferators activated receptors α (PPARα) to upregulate the expression of UCPs. This study also reported an increase in HIF-1α in response to oxidative stress and it was concluded that antioxidant-deficient mitochondria may serve as early cellular signaling responsible for the activation of the Warburg Effect in cancers.

An observation that contributed to the biochemical description of ferroptosis involves the ability of erastin and RSL3 to selectively inhibit the growth of Ras-mutant cancers by inducing a non-apoptotic iron-dependent regulated cell death. It is therefore conceivable that this mutation drives a metabolic reprogramming that makes tumors selectively susceptible to ferroptosis. RSL3 inhibits GPX4, the key antioxidant enzyme that prevents the onset of ferroptosis while erastin is a voltage-dependent anion channels 2 and 3 (VDAC2/3) and cystine-glutamate antiporter (xCT) inhibitor [2]. Mutation in the RAS family of small GTPases (HRAS, NRAS, and KRAS) affects nearly 30% of tumors [1]. Among these, KRAS-driven cancers are characterized by upregulated glycolysis, glutaminolysis, and nucleotide biosynthesis [57]. Upregulation of nucleotide biosynthesis and glutaminolysis suggests the Warburg effect and an active de novo pyrimidine nucleotide biosynthesis which requires glutamine metabolism to provide aspartate [34, 35]. This assertion is supported by the dependence of mutant K-RAS-driven cancers on DHODH [41]. The finding that xCT inhibitors only induce ferroptosis in a fraction of tumor cells sufficiently expressing solute carrier family 7 member 11 (SLC7A11) suggests that in addition to their effect on the functionality of the antioxidant enzyme system, there could be other biochemical mechanisms for ferroptosis induction [58]. It could also imply that to successfully induce ferroptosis, the metabolic phenotype of the tumor must closely resemble that of RAS-mutant cancers where ferroptosis was first induced through SLC7A11 inhibition. Accumulating evidence suggests that one of the metabolic pathways significantly contributing to the sensitivity of RAS-mutant cancers to ferroptosis inducers is de novo pyrimidine nucleotide biosynthesis which under Warburg effect condition, facilitates the shuttling of electrons from complex III to IV.

**Fig. 4:**
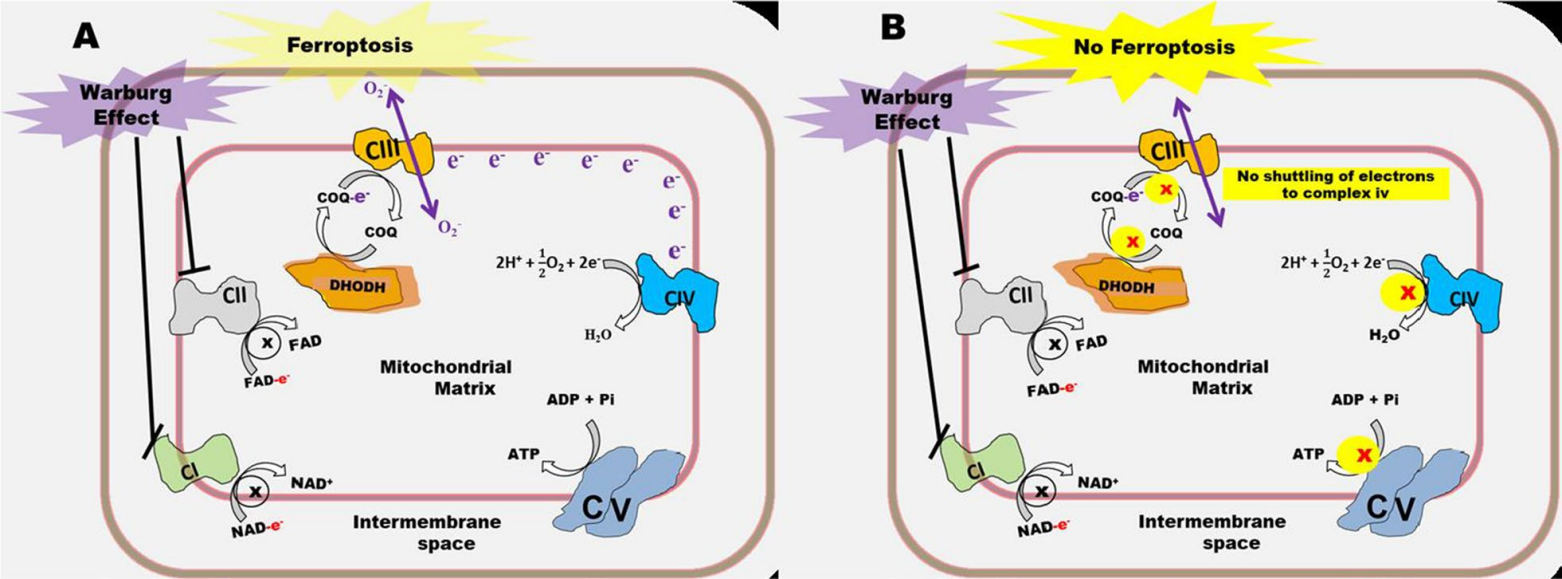
Warburg effect and its modulation of the anti-ferroptosis function of DHODH. **A** In the absence of functional complexes I and II, DHODH sustains the ETC and O_2_^−^ generation by shuttling electrons to complex III. Complex III-generated O_2_^−^ induces oxidative stress that results in ferroptosis. **B** When complexes I and II of the ETC are inhibited by the Warburg effect, DHODH inhibition could cause a complete shutdown of the ETC resulting in the failure of complex III to produce O_2_^−^. Differences between A and B has been highlighted in yellow

Oxidation of CoQ10H_2_ generated by DHODH to CoQ10 by the ETC could result in complex III-mediated generation of O_2_^−^ even when the functionality of complex I and II are defective due to the Warburg effect. Generation of O_2_^−^ by complex III could be required for erastin-induced ferroptosis [13] and possibly, ferroptosis induction in general. Therefore under this condition, if DHODH is inhibited, ferroptosis could equally be inhibited (Fig. 4A and B). The indispensability of a functional DHODH and complex III for tumor growth and the inhibition of complex I and II of the ETC in most tumors by the Warburg effect suggests that under the Warburg effect condition, DHODH-catalyzed reaction could play a significant role in ETC function, O_2_^−^ generation, and ferroptosis induction. It is therefore conceivable that in addition to DNA synthesis which consumes cellular GSH, the Warburg effect could affect the anti-ferroptosis role of DHODH.

## Conclusion and future perspective

**Fig. 5:**
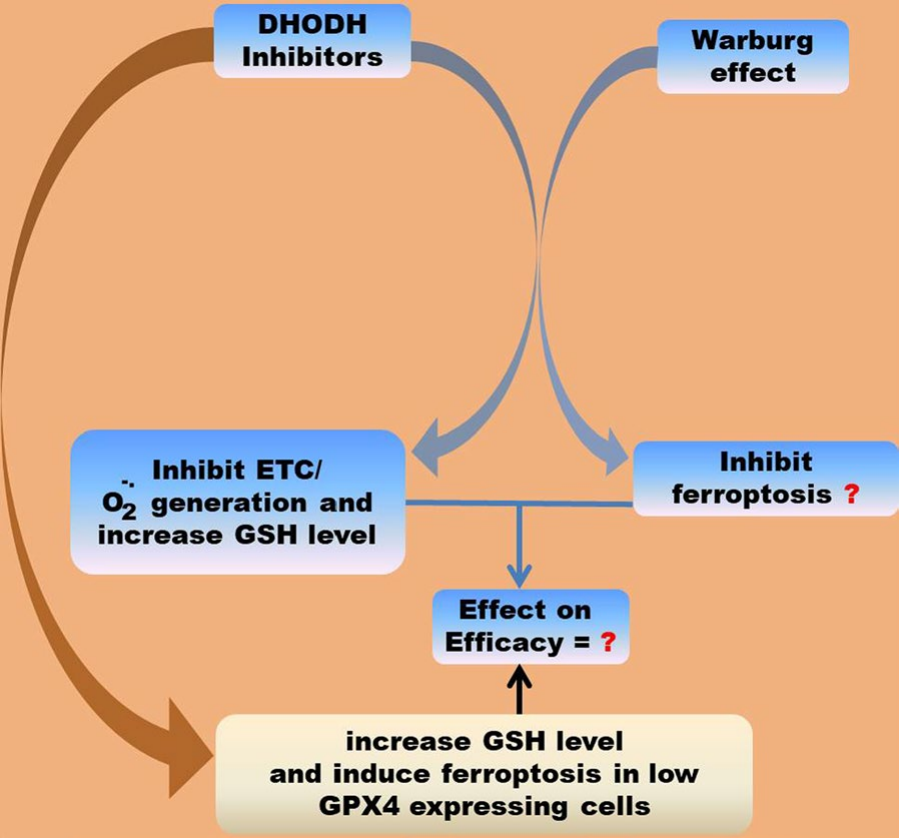
Schematic representation of conclusion and future perspective. As a result of their potential effect on RNR-catalyzed reaction, a reaction that uses GSH to convert NTPs to dNTPs for DNA synthesis, DHODH inhibitors could increase the intracellular level of GSH. Furthermore, under Warburg effect condition, they could inhibit the ETC and O_2_^−^ generation. How this affect the onset of ferroptosis and drug efficacy needs to be investigated

DHODH is a versatile drug target involved in the clinical management of many diseases. It is also an attractive drug target in cancer management. As a result of the importance of de novo pyrimidine nucleotide biosynthesis for tumor growth, an attempt has been made to suppress tumor progression using DHODH inhibitors. One such drug, however, showed unsatisfactory efficacy in phase I and II clinical trials [59, 60]. The role of DHODH in ferroptosis inhibition suggests that its inhibitors could have two complementary mechanisms of action for the suppression of tumor growth that is; inhibition of nucleotide biosynthesis and induction of ferroptosis. However, reports that revealed the indispensability of DHODH-catalyzed reaction for tumor growth, the prevalence of the Warburg effect in solid tumors, and the importance of complex III-generated O_2_^−^ for ferroptosis induction suggests that under Warburg effect condition, DHODH could be responsible for the generation of O_2_^−^ by complex III hence making its inhibition to result in suppressing O_2_^−^ generation and ferroptosis. Inhibiting DHODH could also increase cellular GSH levels thereby making GPX4 to be more efficient in inhibiting ferroptosis. There is therefore a need to investigate the relationship between DHODH inhibitors and how they modulate ferroptosis in solid tumors and how that affects their efficacy. The conclusion and future perspective is schematically represented by Fig. 5.

## Data Availability

Not applicable.

## Abbreviations

GPX4: Glutathione peroxidase 4
DHODH: Dihydroorotate dehydrogenase
ETC: Electron transport chain
GSH: Reduced glutathione
RSL3: Ras-selective lethal compound 3
xCT: Cystine-glutamate antiporter
ATP: Adenosine triphosphate
ROS: Reactive oxygen species
S3QEL: Selective inhibitor of superoxide production from the outer Q-binding site of mitochondrial respiratory complex site III
S1QEL: Selective inhibitor of superoxide production from the outer Q-binding site of mitochondrial respiratory complex site I
KO: Knock out
FCCP: Carbonyl cyanide 4-(trifluoromethoxy) phenylhydrazone
E1: Alpha 1
E2: Alpha 2
OCR: Oxygen consumption rate
NADH: Reduced nicotinamide adenine dinucleotide
NAD^+^: Oxidized nicotinamide adenine dinucleotide
FADH_2_: Reduced flavin adenine dinucleotide
CoQ10: Ubiquinone
CoQ10H_2_: Ubiquinol
FSP1: Ferroptosis suppressor protein 1
DNA: Deoxyribonucleic acid
NTPs: Nucleosides triphosphates
dNTPs: Deoxynucleosides triphosphates
RNR: Ribonucleotide reductase
GSSG: Oxidized glutathione
AML: Acute myeloid leukemia
AOX: Alternative oxidase
mtDNA: Mitochondrial DNA
GOT1: Glutamate–oxaloacetate transaminase 1
MDH2: Malate dehydrogenase 2
GOT2: Glutamate–oxaloacetate transaminase 2
UMP: Uridine monophosphate
TMP: Thymidine monophosphate
dTMP: Deoxythymidine monophosphate
SLC1A3: Solute carrier family 1 member 3
UVB: Ultraviolet B
SDH: Succinate dehydrogenase
FH: Fumarate hydratase
IDH: Isocitrate dehydrogenase
HIF1-α: Hypoxia inducible factor 1 alpha
ODD: Oxygen-dependent degradation domain
PHDs: Prolyl-hydroxylases
NOX: NADPH oxidase
MnSOD: Manganese superoxide dismutase
PINK1: PTEN-induced kinase 1
FOXO3a: Forkhead box O3a
SOD2: Superoxide dismutase 2
M2-PK: M2 isoform of pyruvate kinase
PEP: Phosphoenolpyruvate
NDUFA10: NADH:ubiquinone oxidoreductase subunit A10
AMPK: Adenosine monophosphate kinase
UCPs: Uncoupling proteins
PPAR-α: Peroxisome proliferators activated receptors α
VDAC2/3: Voltage-dependent anion channels 2 and 3
SLC7A11: Solute carrier family 7 member 11
